# Shared historical refugia and genetic diversity hotspots of co-distributed species on the Qinghai Tibet plateau

**DOI:** 10.1016/j.isci.2025.114318

**Published:** 2025-12-02

**Authors:** Hongrui Lv, Dezhi Zhang, Yilin Chen, Yalin Cheng, Deyan Ge, Yanhua Qu, Fumin Lei

**Affiliations:** 1 State Key Laboratory of Animal Biodiversity Conservation and Integrated Pest Management, Institute of Zoology, Chinese Academy of Sciences, Beijing 100101, China; 2 College of Life Sciences, University of Chinese Academy of Sciences, Beijing 100049, China; 3 College of Life Science, Hebei Normal University, Shijiazhuang 050024, China

**Keywords:** Ecology, Wildlife genetics, Zoology

## Abstract

The Qinghai-Tibet Plateau (QTP) is a global biodiversity hotspot, yet the distribution of genetic diversity hotspots in co-distributed species remains poorly understood. Here, we integrate genomic data from three co-distributed endemic species (plateau pika and two snowfinches) on the QTP, with ecological niche models (ENMs) based on paleoclimate data to identify shared genetic diversity hotspots. Our results reveal a consistent northeast-to-southwest decline in genetic diversity, with a shared hotspot located in the northeastern QTP. ENMs further suggest that these species shared glacial refugia in this region during the last glacial maximum, followed by post-glacial expansions into the central plateau, resulting in similar spatial patterns of genetic diversity. Differences in genetic differentiation, demographic history, inbreeding depression, and genetic load suggest species-specific evolutionary responses to historical climate fluctuations. Overall, our study highlights the importance of protecting hotspots of shared genetic diversity across multiple species to preserve their evolutionary potential.

## Highlights

• The northeastern QTP is a shared genetic diversity hotspot for the three species
• Genetic diversity decreasing from northeast to southwest across the QTP
• Species-specific evolutionary responses to historical climate fluctuations
• Conservation priority should focus on northeastern QTP to safeguard evolutionary potential

## Graphical abstract

**Figure:**
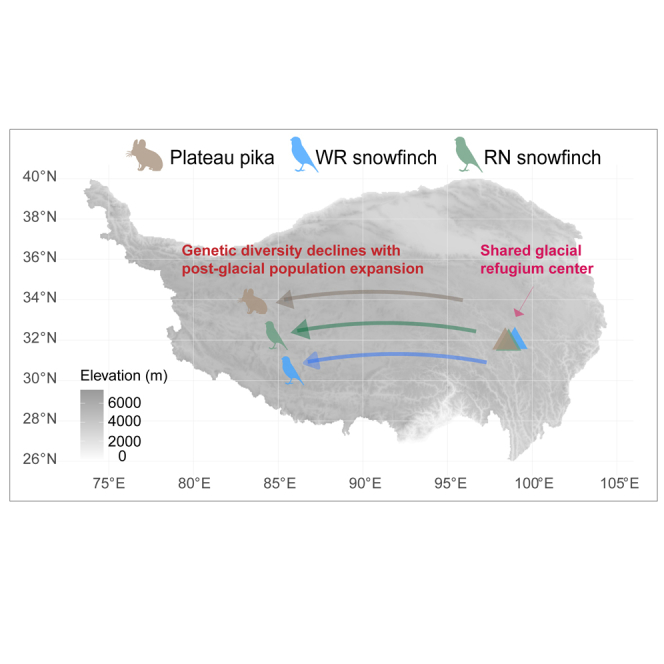

## Introduction

The Qinghai-Tibet Plateau (QTP) is the highest and most ecologically unique alpine ecosystem in the world.^1^^,^^2^^,^^3^ It features a unique geological history, complex climate, diverse habitats, and distinctive landforms.^4^^,^^5^^,^^6^ These factors have driven the diversification and endemism of plateau species,^7^^,^^8^^,^^9^ making the QTP a globally important region for biodiversity conservation.^10^^,^^11^ Since the Quaternary,^12^ dramatic climatic oscillations have driven multiple adaptive radiation events in QTP-endemic lineages,^13^^,^^14^^,^^15^ profoundly influencing and reshaping species distribution patterns and diversification processes.^16^^,^^17^^,^^18^^,^^19^ Notably, over the past few decades, the mean annual temperature in the region has risen at more than twice the global average rate.^20^ This accelerated warming has significantly increased the vulnerability of sensitive alpine ecosystems, especially alpine meadows, leading to ecological risks such as vegetation degradation, habitat loss, and declining species diversity.^21^^,^^22^ In addition, warming-induced upslope tree expansion has exacerbated the habitat loss of endemic flora in the eastern region of the QTP.^23^

Rapid advancements in genomics technology^24^ have provided a robust framework for studying the distribution and diversity of endemic species on the QTP under historical and current climate fluctuations.^25^ Population genetics analyses based on limited molecular markers have revealed that the formation of the diversity of endemic bird species on the QTP correlates significantly with the three major stages of plateau uplift, as exemplified by the white-rumped snowfinch, (*Onychostruthus taczanowskii*, hereafter referred to as WR snowfinch), the red-necked snowfinch (*Pyrgilauda ruficollis*, hereafter RN snowfinch),^15^^,^^26^ and the ground tit (*Pseudopodoces humilis*).^13^^,^^27^^,^^28^ Similarly, phylogeographic analyses have indicated that extant pikas originated on the QTP approximately 14 million years ago and subsequently dispersed to other parts of Eurasia and North America,^18^ supporting the “Out-of-Tibet” hypothesis. Additionally, endemic species exhibit two distinct refuge patterns during glacial-interglacial cycles: one is the “no divergence” pattern, represented by snowfinches,^15^^,^^26^ characterized by a single-origin refuge; the other is the “north-south divergence” pattern, represented by ground tit,^15^^,^^27^ which possesses two independent refugia. Notably, during the Pleistocene, the eastern edge of the QTP, including both northern and southern margins, served as an important biological refuge for different species.^19^ Moreover, research indicates that endemic birds on the QTP exhibit higher nucleotide diversity in the northeastern region compared to the southwestern region.^14^ This similar spatial distribution of genetic (nucleotide) diversity may be closely associated with refuge selection during glacial periods and post-glacial expansion, reflecting the species' adaptive responses to past climate changes and the dynamic shifts in their habitats. Despite extensive research into the effects of historical climate change on the origin,^9^^,^^29^ speciation,^30^ population differentiation,^31^^,^^32^ and glacial refugia of species on the QTP,^16^^,^^19^ most studies remain focused on single species, single taxa, or localized regions by using limited DNA markers. However, comprehensive studies on co-distributed species remain relatively rare, hindering our understanding of their evolutionary responses and dispersal dynamics during past climate fluctuations.

The unique behavior of plateau pikas and snowfinches sharing burrows while living and breeding separately constitutes an ecological relationship between co-distributed species.^33^^,^^34^^,^^35^^,^^36^ As a keystone species in alpine grassland ecosystems and an ecosystem engineer, the plateau pika (*Ochotona curzoniae*) constructs burrows that serve as ideal nesting sites for lizards and snowfinches. In return, snowfinches contribute to reducing predation risk for pikas through their vigilance behavior.^33^^,^^35^ This ecological mutualism promotes coevolution within shared environments, offering significant insights into multi-species co-adaptation on the QTP.^34^^,^^36^ However, this ecological relationship may face significant challenges as climate change intensifies.^37^^,^^38^^,^^39^ Ecological genomics analysis shows that while there is no apparent genetic divergence among populations of the three endemic and resident species (the plateau pika, WR snowfinch, and RN snowfinch), they collectively face similar climate change risks at the population level, particularly in the southwestern part of the QTP.^40^ Due to maladaptation to climate change and landscape barriers, this risk cannot be alleviated by dispersal to more suitable habitats. Although these three species have been relatively well studied on the QTP and are highly representative in terms of their ecological roles and responses to climate change,^14^^,^^15^^,^^18^^,^^36^^,^^41^ our knowledge of their population demographic history, genetic diversity hotspots, and habitat range changes under historical climates remains limited. This gap not only hampers a deeper understanding of their adaptive potential but also poses a challenge for developing effective conservation strategies under future climate change scenarios.

**Figure 1:**
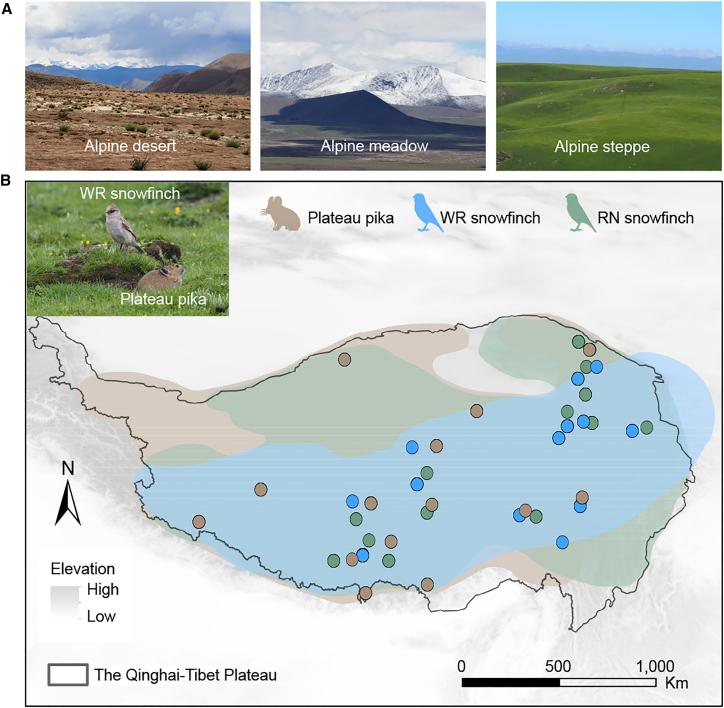
Habitat, distribution, and sampling locations of the three species studied (A) Photos of three habitat types: alpine desert, alpine meadow, and alpine steppe. (B) Distribution and sampling locations of the three species. Different colored dots represent sampling points for each species. The gray polygon represents the QTP. Image was modified from previous study.^40^

In this study, we investigated spatial patterns of genetic diversity for the three species and their response mechanisms to historical climate fluctuations using genome-wide data and paleoclimate-driven ecological niche modeling (Figure 1). Our goals are: 1) to determine whether similar spatial patterns of genetic diversity exist across the three species; 2) to evaluate the potential influence of glacial refugia on genetic diversity and population differentiation; and 3) to examine the demographic history, inbreeding levels, and genetic load of the three species to evaluate their evolutionary responses to past climate fluctuations.

## Results

### Habitat distribution and range shifts across different historical periods

**Figure 2:**
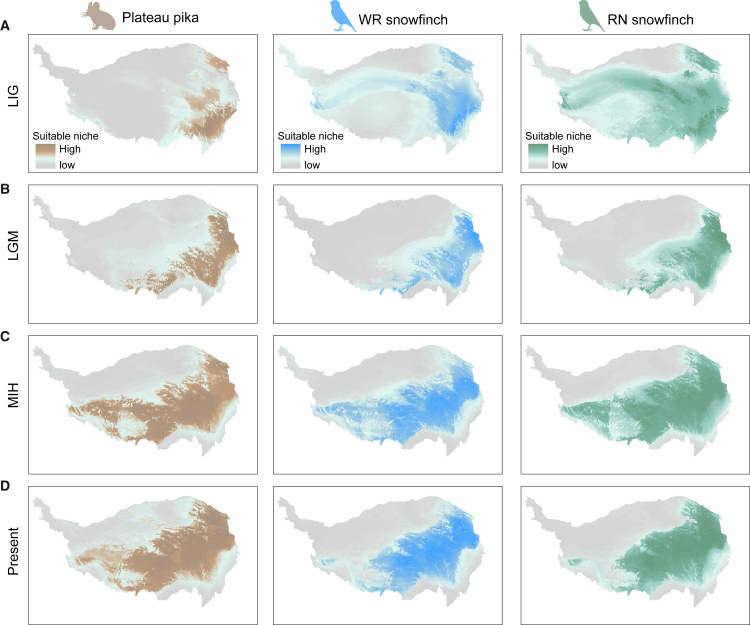
Ecological niche models predicting the suitable niche of the three species (A–D) represent the suitable niche in four historical periods: the LIG (∼140 to 120 ka), the LGM (∼21 to 18 ka), the MIH (∼6 ka), and the present. Warmer colors represent higher distribution likelihoods.

Ecological niche modeling (ENM)^42^ was used to reconstruct suitable habitat for the three species across four periods, including the present, the Middle Holocene (MIH, ∼6 ka), the last glacial maximum (LGM; ∼21 to 18 ka), and the Last Interglacial (LIG, ∼140 to 120 ka). We employed an ensemble modeling approach, averaging projections across different model algorithms in the R package Biomod2.^43^ All ENM had great discrimination ability (true skill statistics [TSS], 0.87–0.897; area under the receiver operating characteristic curve [AUC], 0.978–0.99; Tables S1, S2). The suitable distributions of the three species in the LIG and LGM are located primarily in the eastern and northeastern regions of the QTP (Figures 2A and 2B). From LGM to MIH, the suitable ranges of the three species expanded rapidly, especially in the QTP central region (Figures 2B and 2C), whereas from MIH to the present, there was little change in suitable habitat ranges (Figures 2C and 2D). Overall, the suitable ranges of the three species show consistent performance across historical periods. Refuge populations located in the eastern and northeastern parts of the country expanded rapidly after the recession of the ice age, spreading along the northeast-southwest direction into the central areas of the QTP.

### Genetic diversity decreases with increasing distance from the geographic center of the refuge

**Figure 3:**
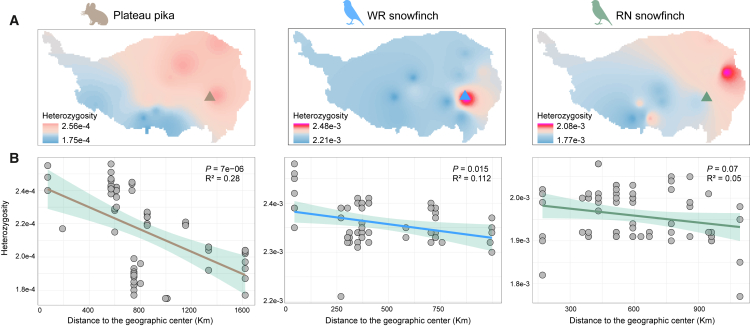
Geographical distribution patterns of genetic diversity (A) Spatial interpolation of genetic diversity (heterozygosity) across the distribution ranges of three species. Dots represent sampling locations for each species. Triangles represent the centers of each species’ geographic distribution during the LGM period. (B) The association between genetic diversity values of all individuals and the geographic center of the refuge. Correlation tests between the two variables were performed using Pearson’s correlation coefficient combined with false discovery rate (FDR) correction and two-tailed tests. The shadow of linear regression denotes the 95% confidence interval.

To assess spatial patterns of genetic diversity across the QTP, we calculated individual genomic heterozygosity for each species and applied kriging interpolation based on these values.^44^^,^^45^ All three species exhibited a general decline in genetic diversity from the northeastern to the southwestern regions of the QTP (Figures 3A and S1, Table S3). Higher diversity was concentrated in northern Qinghai, western Sichuan, and southeastern Gansu, whereas lower diversity levels were observed in southeastern Tibet (e.g., Shigatse and Shannan). We further evaluated the relationship between genetic diversity and distance to refugia during the LGM. The LGM was chosen since it represents the period of habitat compression for species under extreme climate change and had the most significant impact on genetic diversity.^16^^,^^19^^,^^46^ ENMs predicted overlapping LGM refugial areas for the three species in the northeastern QTP. Geographic centers of these refugia were closely located (Figures 3A and S2, Table S4). Across all species, genetic diversity decreased with increasing distance from the refugial center, indicating a consistent spatial signature shaped by historical climate dynamics (Figure 3B).

### Genetic differentiation

We further examined genome-wide genetic differentiation patterns. A strong isolation-by-distance (IBD) signal was detected in plateau pika, with genetic distance between sites (*F*_ST_/(1 - *F*_ST_)) significantly correlated with geographic distance (Mantel’s r = 0.394, *p* = 0.028). In contrast, such a pattern was absent in both snowfinch species (WR snowfinch: r = −0.01, *p* = 0.48; RN snowfinch: r = 0.009, *p* = 0.45).

### Demographic history

**Figure 4:**
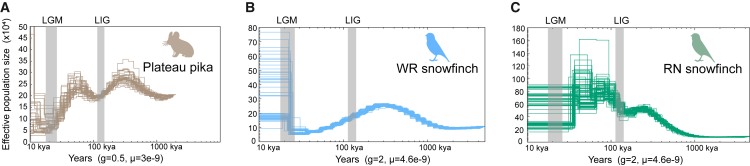
Demographic history of the three species (A) The plateau pika. (B) WR snowfinch. (C) RN snowfinch. PSMC reconstruction of the past demographic history was performed using 100 bootstraps. LGM, and LIG represent the last glacial maximum and the last interglacial, respectively.

Pairwise Sequential Markovian Coalescent (PSMC)^47^^,^^48^ was used to infer the long-term historical demography. The effective population size (*N*_e_) of the plateau pika (Figure 4A) rapidly increased after the LIG,^41^ then gradually declined, reaching its lowest point during the LGM, before rapidly increasing again. The *N*_e_ of WR snowfinch (Figure 4B) gradually decreased after the LIG, dropped to its lowest point during the LGM, and then increased sharply. In contrast, the *N*_e_ of the RN snowfinch (Figure 4C) gradually increased after the LIG and remained relatively stable during the LGM.

### Inbreeding depression

**Figure 5:**
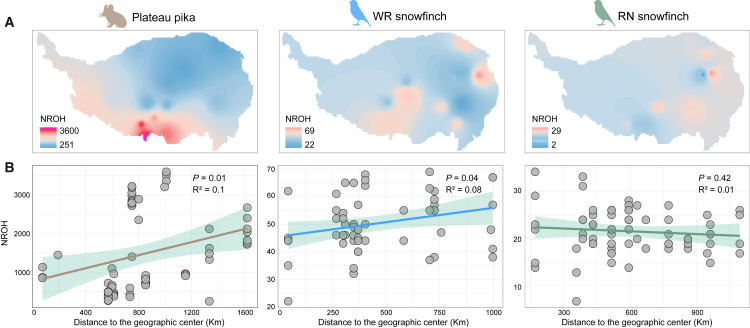
Geographical distribution patterns of inbreeding depression (A) Spatial interpolation of the numbers of ROH across the distribution ranges of the three species. (B) The association between the number of ROH and the geographic center of refuge.

Runs of homozygosity (ROH), as an effective measure of genome-wide homozygosity burden, represent genomic signatures of ancient inbreeding events in historical populations. Over evolutionary time, the original long ROH has gradually shortened due to recombination events.^49^^,^^50^ Based on the generation time of each species and the formula for calculating ROH,^49^ we estimated the ROH length (5 kb - 1 Mb) for each species and mapped the number of ROH to the QTP using spatial interpolation. We found that in southeastern Tibet, the number of ROH was higher in plateau pikas and WR snowfinch, while the number of ROH was lower in RN snowfinch (Figure 5A, Table S3). The number of ROH significantly increased with increasing distance from refuge centers for plateau pika and WR snowfinch, but decreased for RN snowfinch (Figure 5B).

### Genetic load

Generally, genetic diversity reflects the adaptive potential of a species or population, while genetic load is an indicator of maladaptation.^51^ After whole-genome SNP variants were classified into different coding categories^52^: synonymous/nonsynonymous mutations and loss-of-function mutations (LOF), we measured three metrics of genetic load (Table S3). The first is the non-synonymous polymorphic site ratio, *P*_n_/(*P*_n_+*P*_s_), which reflects the accumulation of non-synonymous mutations in the coding region, with higher values indicating that deleterious mutations are more likely to be retained, which may slow down or limit adaptive population evolution.^53^^,^^54^ The second metric is the ratio of the number of nonsynonymous to synonymous polymorphic SNPs, multiplied by the ratio of derived allele frequencies (*P*_n_*f*_n_/*P*_s_*f*_s_), which captures changes in the site frequency spectrum of presumably deleterious alleles relative to the spectrum of presumably neutral alleles.^54^^,^^55^ The increase of *P*_n_*f*_n_/*P*_s_*f*_s_ reflects higher load, with increased genetic drift. The third is the number of LOF mutation,^56^ which reflects the load of mutations that may disrupt gene function and may affect fitness or evolutionary adaptations. To visually observe the distribution of genetic load across the QTP, we performed spatial interpolation using these three metrics.

**Figure 6:**
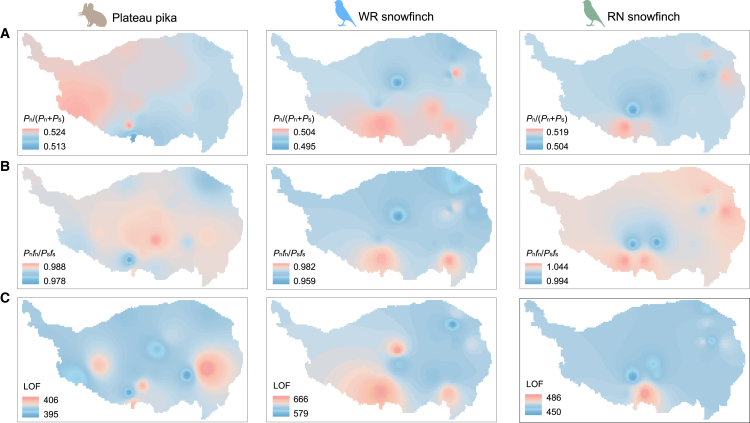
Geographical distribution patterns of genetic load (A–C) Spatial interpolation of genetic loads across the distribution ranges of the three species, calculated through: (A) *P*_n_/(*P*_n_+*P*_s_), (B) *P*_n_*f*_n_/*P*_s_*f*_s_, and (C) the number of LOF.

In the plateau pika, the three genetic load indicators showed an inconsistent geographical distribution pattern (Figures 6A–6C). The areas with high *P*_n_/(*P*_n_+*P*_s_) were mainly located in the southwestern region, showing a decreasing trend from southwest to northeast; *P*_n_*f*_n_/*P*_s_*f*_s_ reached high values in the central part, exhibiting characteristics of being high in the center and low at the edges; and the distribution of the LOF mutations was relatively fragmented, occurring in the eastern and southeastern regions. In contrast, for both snowfinches, the three genetic load indicators showed a consistent geographical distribution pattern (Figures 6A–6C), with high load areas mainly concentrated in the southern, southeastern, and partly northeastern regions. We also analyzed the associations of the three metrics for each species with geographic centers of refuge, and found significant positive associations only for *P*_n_/(*P*_n_+*P*_s_) in the plateau pika, but not in any of the other metrics (Figure S3).

## Discussion

Understanding how co-distributed species respond to historical climatic fluctuations is critical for elucidating evolutionary processes in alpine ecosystems and informing effective conservation strategies. By integrating population genomics with ENMs, our study revealed that plateau pikas and two snowfinches may have shared glacial refugia during the LGM and subsequently underwent postglacial expansions. This dynamic evolutionary process likely shaped their northeast-to-southwest genetic diversity gradient, forming a high diversity hotspot in the northeastern QTP. While the three species showed similar spatial gradients, we still observed differences in genetic differentiation, inbreeding depression, and genetic load, reflecting different evolutionary responses to similar environmental histories. Overall, our findings provide new insights into the evolutionary dynamics of alpine species and important scientific evidence for effective conservation under future climate change.

### Shared glacial refugia and post-glacial expansion

**Figure 7:**
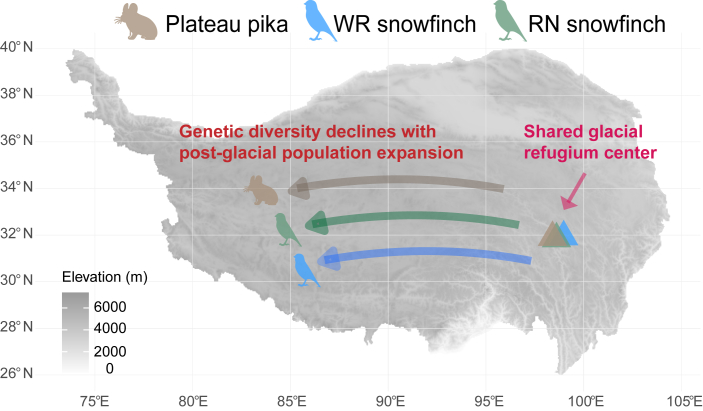
Genetic diversity declines with post-glacial population expansion after the LGM The triangles represent the centers of refugia in the LGM for the three species.

Glacial refugia and postglacial dispersal played a crucial role in shaping the genetic diversity and distribution patterns of many species on the QTP.^16^^,^^19^ We found that during the LGM, the northeastern region served as the glacial refugia for these three species. This finding not only aligns with previous studies,^14^^,^^15^^,^^27^^,^^41^ but also corresponds with the refugial distribution patterns inferred for some plants^57^ and insects.^58^ For example, three birds, the Tibetan snowfinch (*Montifringilla adamsi*), the Blanford’s snowfinch (*Pyrgilauda blanfordi*), the horned lark (*Eremophila alpestris*), used the eastern margin of the QTP as their primary refuge during the Pleistocene glacial period.^14^ Notably, we found that the refugial centers of the three species during the LGM were geographically proximate (Figure 7), implying that they may have experienced similar processes of habitat contraction and co-occurrence maintenance during this period.

Although some studies have shown that snowfinches and plateau pika do not form a true mutualistic relationship, with their interactions mainly limited to snow finches issuing warning calls when predators appear, the dependence of snowfinches on pika burrows is crucial to their survival under the harsh conditions of the QTP.^34^^,^^36^ As an “umbrella species”, plateau pikas provide essential nesting sites for snowfinches, thereby enhancing their ability to cope with historical climatic fluctuations (Figure 7). These burrows offer shelter, especially during extreme weather events such as heavy rain, hail, and strong winds. Many birds on the plateau rely on plateau pika burrows for nesting, and a decline in plateau pika populations or the destruction of their burrows can significantly reduce the numbers of these birds, highlighting the critical importance of this asymmetric ecological dependence.^34^^,^^36^ In contrast, the ground tit, with its long, curved bill, was able to excavate its own burrows, making it less reliant on those constructed by plateau pikas.^28^^,^^59^ This behavioral divergence, together with climatic and genetic factors, may explain the differences in demographic history and dispersal dynamics among species.

Based on the closeness of the glacial refugia centers for the three species, we further inferred their dispersal routes during the postglacial period. The plateau pika and two snowfinches may have dispersed along similar pathways from the northeast to the central QTP after the LGM (Figure 7). Largely consistent with the dispersal patterns observed in plateau pikas and snowfinches, analyses of historical refugia and gene flow suggest that the three QTP birds (*M*. *adamsi, P*. *blanfordi,* and *E*. *alpestris*) mentioned above likely followed similar postglacial dispersal routes, expanding from the eastern edge to the central region of the QTP.^14^ While climate change is a key factor in shaping the geographic distribution of species,^10^^,^^27^ ecological relationships between species also play an important role.^34^^,^^35^^,^^36^ Therefore, our findings highlight the importance of ecological relationships and climate change in shaping the biogeographic patterns of co-distributed species.

### The northeastern QTP: A crucial hotspot of genetic diversity

Genetic diversity is a fundamental determinant of a species’ evolutionary potential and resilience to environmental change.^60^^,^^61^ The northeastern region of the QTP is not only an important center of the postglacial expansion of many alpine animals and plants,^16^^,^^19^ but also a hotspot of genetic diversity.^62^ Our results reveal that the hotspot of high genetic diversity shared by the three species is in the northeastern region, largely overlapping with their inferred glacial refugia. This area is likely a key source of post-glacial expansion, providing support for population recovery and long-term genetic diversity. After the LGM, the genetic diversity of these three species gradually decreased as they expanded together from the northeast region into the central region (Figure 7). Populations at the expansion front, particularly in southeastern Tibet (e.g., Shannan and Nyingchi), exhibited markedly lower genetic diversity. Although this pattern was evident in plateau pikas, it was less pronounced in the two snowfinches. The genetic diversity of plateau pikas and RN snowfinches showed a gradual decline from northeast to southwest, whereas in WR snowfinches, genetic diversity was significantly higher only in parts of the northeast region and lower elsewhere. We suggest that these patterns may be attributed to differences in migration ability, demographic history, and ecological adaptability of the three species. Taken together, these findings provide new insights into the genetic responses of co-distributed species to climate change and lay the foundation for further exploration of their ecological relationships and evolutionary dynamics.

### Differences in population dynamics and evolutionary responses across the three species

Climate change has played a key role in shaping the population history and geographical dispersal of alpine species, yet responses to similar climatic stresses may vary significantly across species.^63^^,^^64^ Our findings further suggest that the plateau pika and the two snowfinches may have evolved distinct adaptive responses to similar historical climatic fluctuations. The PSMC results (Figure 4) show that the *N*_e_ of the plateau pika increased rapidly after the LIG, but declined to a minimum during the LGM, after which it recovered rapidly.^41^ This suggests that the plateau pika has experienced a population bottleneck during the LGM, and then expanded and increased rapidly as the glacier receded. As the distance from the LGM refugia center increased, plateau pikas showed increased genetic differentiation, a significant decrease in genetic diversity, and an increase in the number of ROH, which indicates increased genetic drift and restricted gene flow. The WR snowfinch also displayed a demographic history that broadly mirrored that of the plateau pika. Due to its high migratory ability, the WR snowfinch exhibits relatively little genetic differentiation and fewer constraints on gene flow.^40^ Notably, with distance increasing from LGM refuge centers, WR snowfinches show a significant increase in the number of ROH and an increase in inbreeding, leading to reduced genetic diversity. This indicates that the WR snowfinch experienced a relatively slow-paced range expansion in response to climate fluctuations, accompanied by significant genetic drift. By contrast, gene flow is less restricted in RN snowfinch,^40^ whose *N*_e_ gradually increased after the LIG and remained stable during the LGM. As the distance increasing from LGM refuge centers, the number of ROH decreases slightly, inbreeding slightly decreased, and genetic diversity decreases, indicating that this species may have mitigated the effects of inbreeding through continuous gene flow during expansion, but are still affected by the founder effect. In summary, these species showed different patterns of genetic response and dispersal dynamics in the face of similar climatic stresses, reflecting the complex mechanisms by which climate change, demographic history and ecological relationships combine to shape the adaptive evolution of alpine species.

This study emphasizes the importance of integrating ecological relationships and climate change into multi-species conservation. Similar patterns of distribution and genetic diversity among multiple species highlight the need to conserve co-distributed species and their key ecological relationships, especially keystone species such as the plateau pika,^34^^,^^36^ which are critical to the long-term survival of other species. The northeastern part of the QTP is particularly valuable as a hotspot of high genetic diversity shared by the three species, which is key to maintaining the long-term evolutionary potential of the plateau species. In conclusion, our study has initially provided valuable insights for the conservation of mountain ecosystems and their co-distribution animal species, particularly conservation strategies for genetic diversity and evolutionary potential in the context of future climate change scenarios.

### Limitations of the study

However, this study also has limitations due to its present bias in sampling and other aspects. First, we did not consider the suitable distribution areas of the plateau pika outside the QTP, which may affect our understanding of its historical distribution ranges.^16^^,^^36^ Second, high-quality genomic data can provide additional information on genetic variation, including insertions and deletions,^65^ and other structural variations,^66^ which have not been considered in this study. Additionally, we primarily focused on the impact of climate fluctuations and did not consider other ecological factors, such as interspecies competition^67^ and predation pressure,^68^ which may also play significant roles in species evolution.

## Resource availability

### Lead contact

Further information and requests for resources should be directed to Prof. Fumin Lei (leifm@ioz.ac.cn).

### Materials availability

This study did not generate new unique materials.

### Data and code availability

• No new genomic data were generated in this study. All genomic and distributional data used were obtained from a previous study.^40^
• This article does not report original code. Any additional information required to reanalyze the data reported in this study is available from the lead contact upon request.

## Acknowledgments

We thank the guest editors, Fuwen Wei and Li Yu, for the invitation to contribute this article to the special issue: “Conservation Genomics and Metagenomics.” This study was supported by grants from the 10.13039/100014718National Natural Science Foundation of China (32130013 to F.L.), the 10.13039/501100012166National Key Research and Development Program of China (2022YFC2601601 to F.L.), and the grant from the 10.13039/501100011186Institute of Zoology, Chinese Academy of Sciences (2023IOZ0104 to F.L.).

## Author contributions

Fumin Lei and Hongrui Lv designed and supervised the study; Hongrui Lv, Dezhi Zhang, and Yilin Chen cosupervised the study; Hongrui Lv conducted data analysis and interpretation; Hongrui Lv, Dezhi Zhang, Yilin Chen, Yalin Cheng, Deyan Ge, Yanhua Qu, and Fumin Lei contributed to writing the article.

## Declaration of interests

The authors declare no competing interests.

## STAR★Methods

### Key resources table

**Table:** 

REAGENT or RESOURCE	SOURCE	IDENTIFIER
**Software and algorithms**		

PLINK v1.9	Purcell et al.^69^	https://www.cog-genomics.org/plink2/
GATK	McKenna et al.^70^	https://gatk.broadinstitute.org/hc/en-us
VCFtools v.0.1.13	Danecek et al.^71^	https://vcftools.github.io/
PSMC	Li and Durbin^47^	https://github.com/lh3/psmc
ANNOVAR	Wang et al.^52^	https://annovar.openbioinformatics.org/en/latest/
R v4.X	R Core Team	https://www.r-project.org/
Biomod2 package	Thuiller et al.^43^	https://github.com/biomodhub/biomod2
gstat package	Gräler et al.^72^	https://github.com/r-spatial/gstat/
ggplot2 package	Ginestet^73^	https://github.com/hadley/ggplot2-book
geosphere package	Hijmans^74^	https://github.com/rspatial/geosphere
vegan package	Dixon^75^	https://github.com/vegandevs/vegan
terra package	Hijmans^76^	https://rspatial.org/
tmap package	Tennekes^77^	https://github.com/r-tmap/tmap
sf package	Pebesma^78^	https://github.com/r-spatial/sf/

### Experimental model and study participant details

#### Study area

The study was conducted in the QTP, a high-altitude region with diverse climatic and topographic conditions, making it an ideal system to investigate environmental adaptation and ecological divergence.

#### Sample collection

Genomic data was obtained from a previous study,^40^ with 66, 68 and 55 samples for plateau pika, WR snowfinch and RN snowfinch, respectively. Genomic data for WR snowfinch and RN snowfinch individuals are available in the National Center for Biotechnology Information (NCBI) Short Read Archive under project number PRJNA417520 (https://www.ncbi.nlm.nih.gov/bioproject/PRJNA417520) and in the National Genomics Data Center (NGDC) under accession number CNP0004029 (https://db.cngb.org/search/project/CNP0004029/).Genomic data for the plateau pika are accessible from the NGDC under NP0003365 (https://db.cngb.org/search/project/CNP0003365/) and CNP0004029 (https://db.cngb.org/search/project/CNP0004029/). Species occurrence records, also sourced from the same study, with 369, 212, and 250 points for plateau pika, WR snowfinch and RN snowfinch, respectively. The distribution ranges of the three species were obtained from the International Union for Conservation of Nature (IUCN; https://www.iucn.org/), and their distribution ranges cover a major part of the QTP.

### Method details

#### Ecological niche modeling

The ensemble modeling approach in the R package Biomod2^43^ was used to predict range shifts of suitable distribution using bioclimatic variables across four periods: the present, the MIH (∼6 ka), the LGM (∼21 to 18 ka), and the LIG (∼140 to 120 ka). We downloaded 19 bioclimatic variables from WorldClim version 1.4.^79^ To avoid multicollinearity among the 19 environmental variables, we selected 7, 6, and 6 variables for the ENM analysis (Table S2), ensuring that the Spearman correlation coefficients |*r*| < 0.7 for the plateau pika, WR snowfinch, and RN snowfinch, respectively. A total of 10,000 pseudoabsence points (or background points) were randomly sampled across species’ ranges, giving equal weights to presence and background points. Cross-validation was employed with five repeats by randomly splitting occurrence records into two subsets (75% of the data for modeling calibration and the remaining 25% for testing). Based on sensitivity >0.8 and specificity >0.8, we retained 6, 7, and 6 relatively strong models for the plateau pika, WR snowfinch and RN snowfinch, respectively (Table S2). To improve prediction accuracy, models with AUC >0.7 and TSS >0.7 were retained in the ensemble prediction. ENM outputs include species presence (1) and absence (0) predictions.^80^ Maps and spatial visualizations were produced using the R packages ggplot2,^73^ tmap,^77^ and sf.^78^

#### Estimates of genetic diversity and spatial interpolation

Heterozygosity was calculated for each sampled individual using GATK,^70^ which determines the number of heterozygous sites in each genome and calculates the degree of heterozygosity by dividing the total number of heterozygous sites by the sum of homozygous and heterozygous sites.^81^ We used the krige function in the R package gstat^72^ to spatially interpolate the genetic diversity values for each species to observe their geographic distribution on the QTP. We selected habitats as refugia during the LGM and calculated the geographic centers of refugia for the three species. ENM was used to predict the distribution points of each species during the LGM, labeled as present (1) or absent (0). We then selected all distribution points predicted as present (1) by ENM and calculated the geographic centers of these points.

#### Isolation-by-distance test

To investigate whether population genetic differentiation changes with increasing geographic distance, we evaluated the isolation-distance model.^82^ Based on independent locus SNPs, we calculated a matrix of pairwise genetic distances *F*_*ST*_/(1 − *F*_*ST*_) between sampling sites for each species using the R package hierfstat.^83^ Independent locus SNPs were extracted using PLINK v1.90^69^ with the parameters “indep-pairwise 50 10 0.2” to extract an LD-pruned SNP set with a minimum allele frequency (MAF) greater than 10%. Euclidean geographic distances between sampling points were obtained from coordinate data and calculated using the R package geosphere (https://github.com/rspatial/geosphere). We used the R package vegan^75^ to assess the effect of geographic factors on genetic differentiation.

#### Demographic history reconstruction

PSMC^47^ was used to infer historical changes in the effective population size (Ne) using default parameters with the entire genomic dataset. We remapped based on published PSMC results for plateau pikas.^41^ Demographic history reconstruction for all snowfinch individuals was conducted with a generation time of 2 years and a mutation rate of 4.6 × 10^−9^ mutations per generation.^84^ PSMC reconstruction of the past demographic history was performed using 100 bootstraps replicates.

#### Estimates of ROH

ROH were used to assess the level of inbreeding.^49^ We used VCFtools v.0.1.13^71^ to convert the VCF file to PED and MAP formats, and then used PLINK v.1.90 to infer the number and length of ROH. Based on the formula for calculating ROH (g = 100/2∗ROHlength)^50^ and the generation time of each species, we calculated the number of ROH with lengths ranging from 5 kb to 1 Mb. Then, we used the krige function in the R package gstat^72^ to spatially interpolate the number of ROH for each species to observe their geographic distribution on the QTP.

#### Estimates of genetic load and spatial interpolation

The genetic load for each species was calculated by annotating the biallelic SNP sites present in all individuals. All genomic variation sites were annotated in ANNOVAR^52^ software using each species' reference genome,^17^^,^^85^ classifying the variations into three types: synonymous mutations, missense mutations, and loss-of-function (LOF) mutations. We calculated three metrics of genetic load^53^^,^^54^^,^^56^: the non-synonymous polymorphic site ratio, *P*_n_/(*P*_n_+*P*_s_), the ratio of the number of nonsynonymous to synonymous polymorphic SNPs, multiplied by the ratio of derived allele frequencies (*P*_n_*f*_n_/*P*_s_*f*_s_), and the number of LOF. We then also used the krige function in the R package gstat^72^ to spatially interpolate each of the three metrics of genetic load for each species to observe their geographic distribution on the QTP.

### Quantification and statistical analysis

For all population genetic analyses reported here, the following sample sizes were used: plateau pika (*n* = 66), WR snowfinch (*n* = 68), and RN snowfinch (*n* = 55). Correlation tests between the two variables were performed using Pearson’s correlation coefficient combined with false discovery rate (FDR) correction and two-tailed tests.

## Supplemental information

Document S1. Figures S1–S3

Table S1. Sample information and 19 climate variables of the three species

Table S2. Environmental variables, methods and assessment indicators selected in the ENM analysis for the three species

Table S3. Genetic diversity, distance to the refuge center, ROH and genetic load for the three species

Table S4. The refuge centers for the three species during the LGM period
