# Supplementary material for: Shared historical refugia and genetic diversity hotspots of co-distributed species on the Qinghai Tibet plateau
Source: iScience. 2025 Dec 2;29(1):114318. doi: 10.1016/j.isci.2025.114318 (PMC12834105; doi:10.1016/j.isci.2025.114318)
Supplement: Document S1. Figures S1–S3 [file mmc1.pdf]

iScience, Volume *29*

Supplemental information

Shared historical refugia and genetic diversity hotspots of co-distributed species on the Qinghai Tibet plateau

Hongrui Lv, Dezhi Zhang, Yilin Chen, Yalin Cheng, Deyan Ge, Yanhua Qu, and Fumin Lei

Supplementary Information

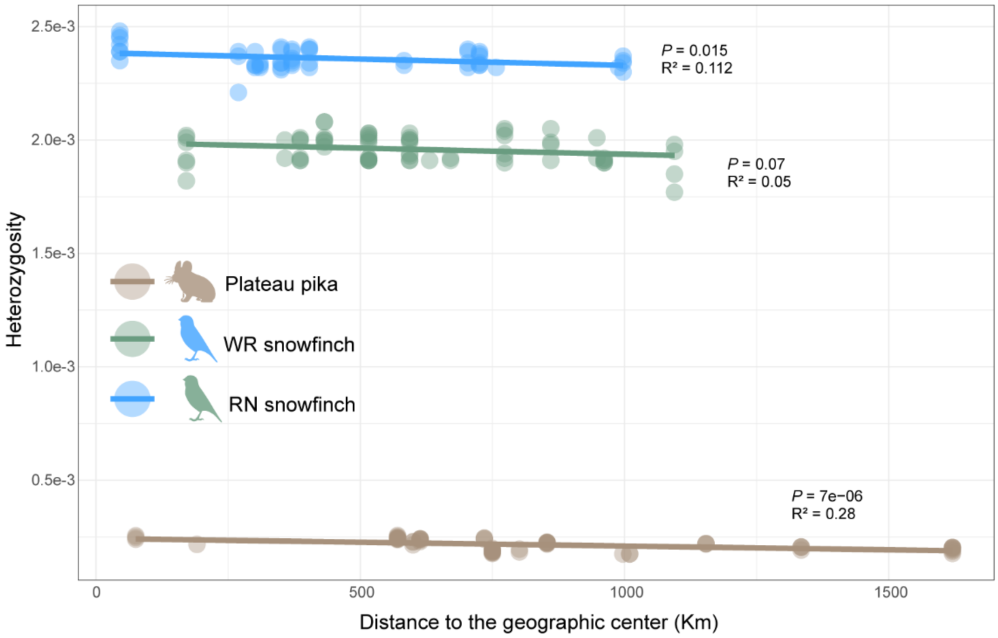

Figure S1 Geographical distribution patterns of genetic diversity across three species.

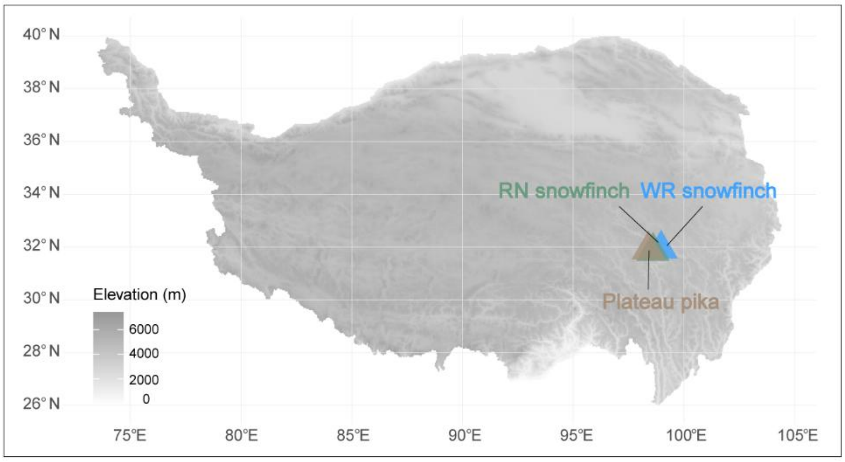

Figure S2 The geographical centers of the refugia for three species during the LGM period.

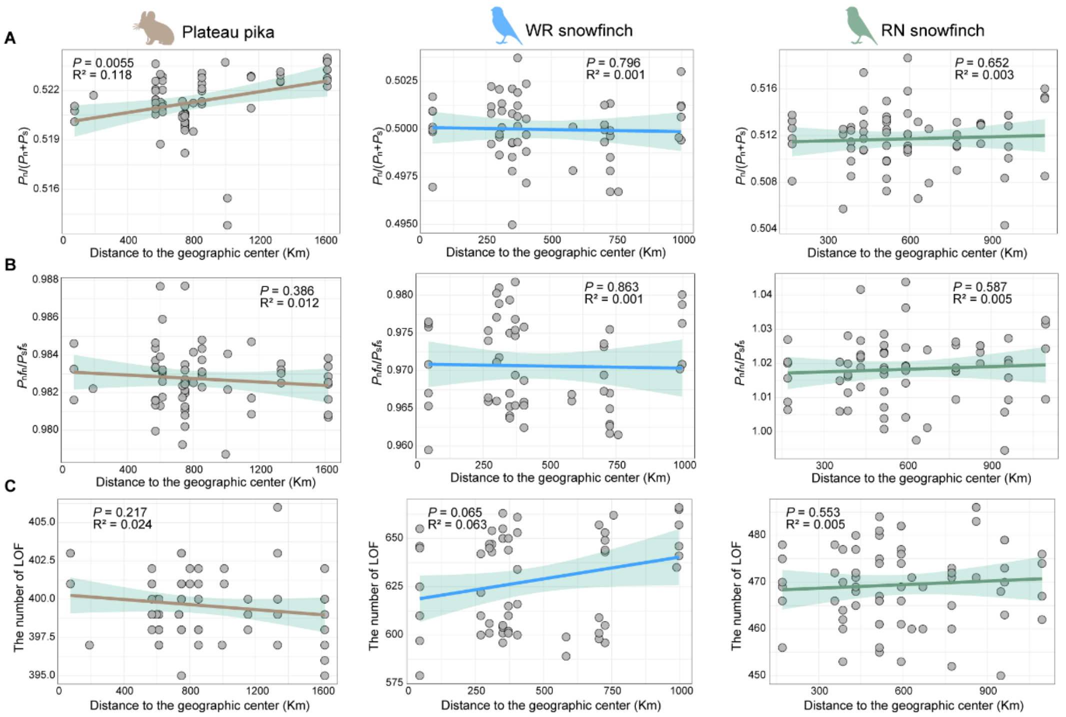

Figure S3 Association between genetic load and distance to geographic centers of refuge for three species. (A) to (C), The association between genetic load and the geographic center of refuge of the three species, calculated through: **(A)** $P_n/(P_n+P_s)$; **(B)** P_nf_n/P_sf_s; **(C)** the number of LOF.
